# Artificial Intelligence for Microbial Colony Analysis: Plate-Reading Challenges, Method Selection, and Validation

**DOI:** 10.3390/microorganisms14102251

**Published:** 2026-10-04

**Authors:** Xue Li, Jing Wang, Meng Xiao, Peiyao Jia, Dingding Li, Yu Hu, Meng Shi, Qiang Xu, Yi Lu, Yingchun Xu

**Affiliations:** 1 Department of Laboratory Medicine, State Key Laboratory of Complex Severe and Rare Diseases, Peking Union Medical College Hospital, Chinese Academy of Medical Sciences and Peking Union Medical College, Beijing 100730, China; 2 Clinical Biobank, Biomedical Engineering Facility of National Infrastructures for Translational Medicine, Chinese Academy of Medical Sciences, Peking Union Medical College Hospital, Beijing 100730, China; 3 State Key Laboratory of Mechanical System and Vibration, School of Mechanical Engineering, Shanghai Jiao Tong University, Shanghai 200240, China; 4 Silliker Food Science Center Mérieux NutriSciences (China), Mérieux Nutrisciences Testing Technology (Shanghai) Co., Ltd., Shanghai 201112, China; 5 Yangtze River Pharmaceutical Group, Taizhou 225321, Jiangsu, China; 6 Jieyi Biotechnology (Suzhou) Co., Ltd. (Fasteur), Suzhou 215122, Jiangsu, China

**Keywords:** colony enumeration, digital microbiology, instance segmentation, urine-culture screening, membrane filtration, method validation

## Abstract

Microbial colony analysis is routinely used in clinical microbiology, pharmaceutical quality control, food safety, and environmental monitoring. Colony enumeration remains challenging because real-world plates frequently show crowding, size variation, background debris, and plate-rim interference. Low contrast on membrane or filter media, weak colony boundaries, and ultra-dense or too-numerous-to-count (TNTC) growth further complicate analysis. These conditions increase counting variability and limit rule-based counters. This review examines artificial intelligence (AI)-enabled colony analysis and links recurrent plate-reading challenges to method selection and validation requirements. We examine traditional threshold- and morphology-based pipelines, object detection, semantic segmentation, instance segmentation, and density- or keypoint-based estimation. Current evidence is stronger for plate-level triage and semi-quantitative interpretation than for direct colony enumeration. However, evidence supporting routine quantitative colony-forming unit (CFU) reporting across laboratories remains limited. Reliable deployment requires methods suited to plate format, organism, imaging conditions, and intended use, with validation in the target workflow.

## 1. Introduction

### 1.1. Colony Enumeration as the Core Task in Microbial Colony Analysis

Colony enumeration on solid media provides quantitative information for routine decisions in clinical diagnostics, pharmaceutical manufacturing, food safety, and environmental surveillance [1,2,3,4]. A visible colony is not necessarily equivalent to one microbial cell: a colony-forming unit (CFU) may originate from a single cell, a chain or clump of cells, or, for filamentous organisms, a viable hyphal fragment. Accordingly, image analysis systems enumerate visible colony objects, whereas the reported CFU concentration remains conditional on sample dispersion, dilution, inoculated volume, culture conditions, and the applicable counting convention. Reliable enumeration also requires colony detection, delineation, discrimination from artifacts, and the interpretation of morphology because colonies on routine plates are not always clearly separated.

In clinical workflows, quantitative urine culture aids in distinguishing infection from contamination and informs triage and treatment decisions [4,5,6,7]. In urine culture, thresholds such as ≥10^5^ CFU/mL have historically been used in specific interpretive contexts for urinary tract infections (UTIs), although current clinical interpretation is more nuanced and depends on specimen type, patient population, organism identity, and associated clinical information. In pharmaceutical quality control (QC), microbial limits and sterility testing are central to product-release decisions and compliance with compendial requirements [8,9,10,11]. In food microbiology, colony counts support hygiene monitoring and pathogen detection (e.g., *Escherichia coli*, *Salmonella* spp.), guided by standardized methods and regulatory expectations [1,10,12,13,14]. Environmental monitoring similarly relies on quantitative culture to assess microbial burden and detect emerging risks in water, air, and healthcare settings [9,15,16,17,18].

### 1.2. Limitations of Manual and Traditional Automated Methods

Manual visual counting is limited by subjectivity and poor scalability [19,20,21]. Inter-operator variability can be substantial and is influenced by examiner experience, visual fatigue, viewing conditions, and differences in interpretation [22,23,24]. These problems become more pronounced on challenging plates, especially those with dense or confluent growth, pinpoint colonies, or species with complex phenotypes. For example, *Proteus* spp. may produce swarming growth, whereas *Pseudomonas aeruginosa* may form large spreading colonies with irregular margins. In addition, diffusible pigments produced by *P. aeruginosa* can alter the color and intensity of the surrounding agar, thereby confounding thresholding and background-subtraction algorithms [25,26,27,28]. As sample volume increases, manual reading can also constrain throughput. It consumes skilled labor, prolongs turnaround time, and increases operating costs [21].

Classical automated counters were introduced to reduce this burden through handcrafted image-processing pipelines, including thresholding, edge detection, and watershed segmentation [25,26,27]. These systems can perform adequately on clean, low-density plates, but their performance often deteriorates under routine conditions. Overlapping or confluent colonies challenge segmentation, and common artifacts such as scratches, bubbles, condensation, debris, uneven illumination, and reflections can lead to false detections [14,26]. Many automated workflows therefore still require substantial human review and correction, reducing their efficiency [23]. Young et al. found that automated counting did not consistently outperform human readers, particularly for dense or overlapping colonies [23]. Heuser et al. reported the limitations of a commercial automated counter under confluent growth and small-colony conditions [26].

### 1.3. The Rise in AI in Microbiological Image Analysis

**Figure 1:**
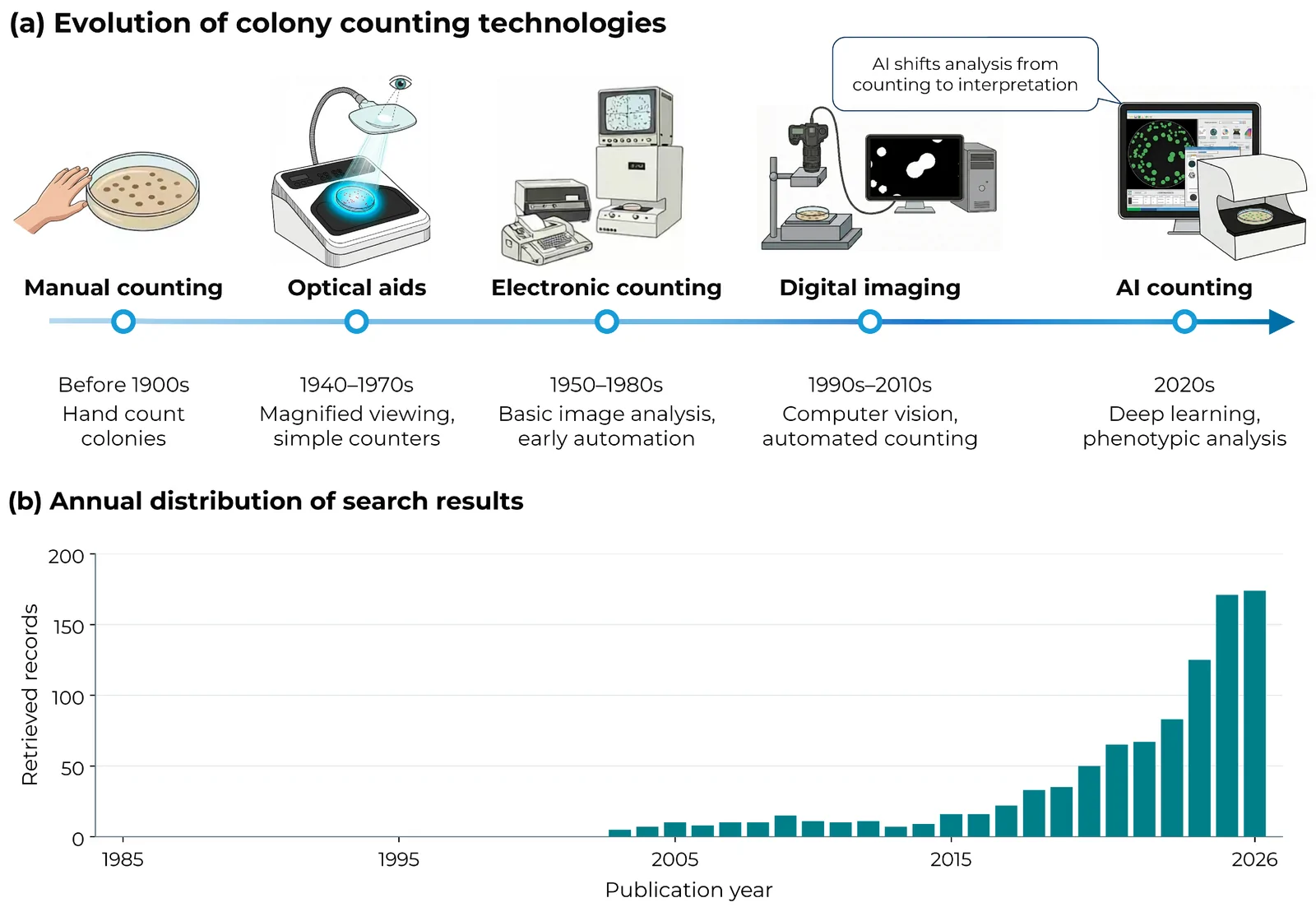
Historical development of colony counting and annual distribution of the updated search results. (**a**) Conceptual progression from manual counting to optical aids, electronic systems, digital imaging, and AI-enabled analysis. The periods overlap and indicate broad stages rather than exclusive adoption dates or the first report of each method; CNN-based colony analysis predates the 2020s. (**b**) Annual distribution of all 970 Web of Science Core Collection records retrieved on 20 September 2026 using the query and editions specified in Section 2.1, without language or document-type restrictions or eligibility filtering. The 2026 count covers only records retrieved by the search date.

Figure 1 summarizes the progression of colony counting from manual inspection to contemporary AI-enabled analysis [25,29,30,31,32,33,34]. Early work established practical counting ranges for human readers, including the empirical limits reported by Breed and Dotterrer in 1916 [29]. Subsequent decades introduced mechanical and electronic aids. Electronic bacterial-cell counting was reported in the late 1950s [30], followed by automated devices for counting visible colonies [31,34]. Digital imaging was subsequently combined with traditional computer-vision algorithms [25]. Convolutional neural network (CNN)-based colony analysis emerged in the mid-2010s and subsequently expanded. These approaches have demonstrated advantages over selected classical baselines in specific datasets and controlled comparisons [33,35,36]. Figure 1b shows the annual distribution of 970 records retrieved from the Web of Science Core Collection using colony-counting and image analysis terms with a publication-year condition of 1985–2026.

**Figure 2:**
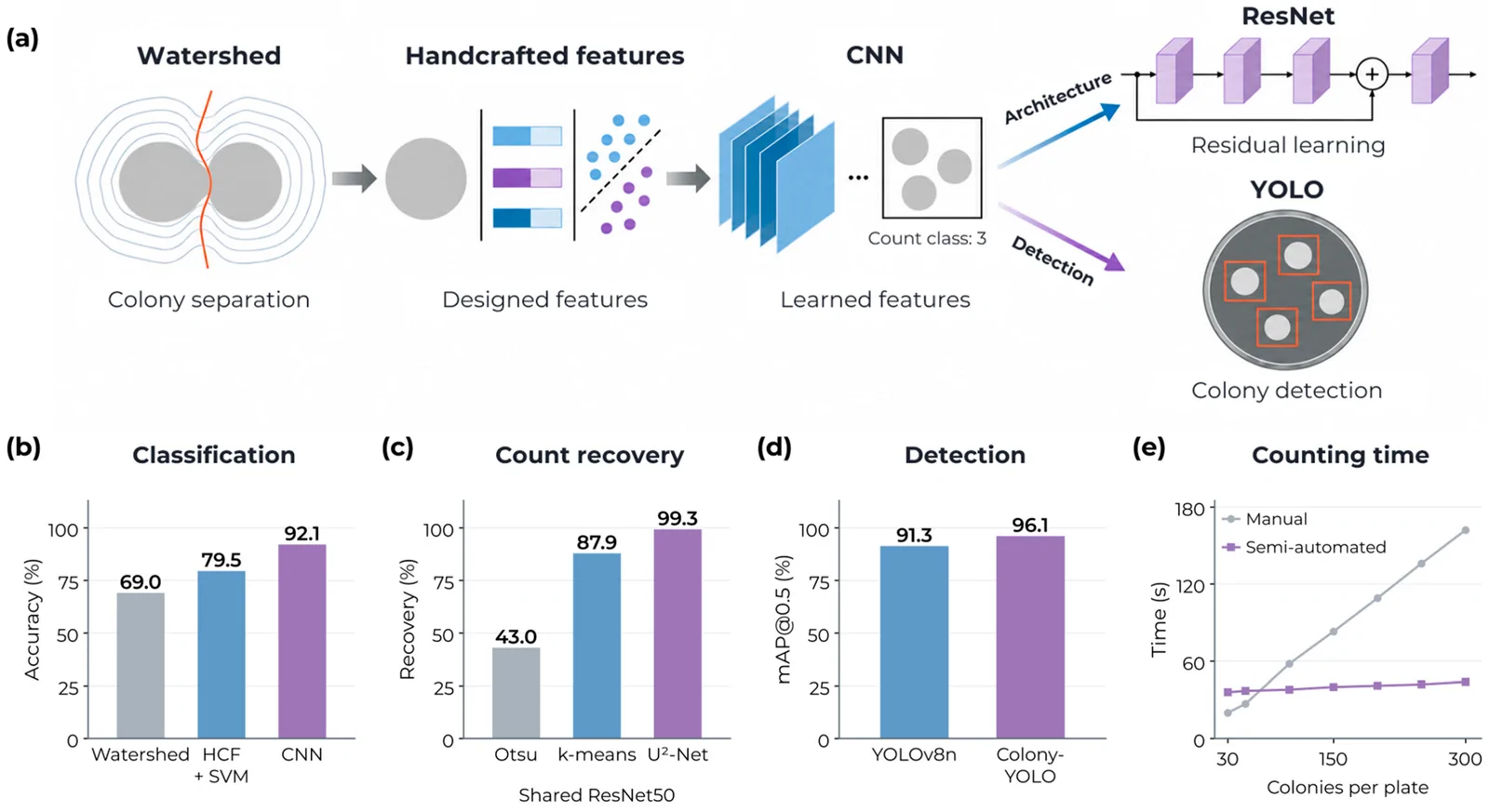
Conceptual relationships among colony-analysis methods and within-study comparisons. (**a**) Developments from watershed separation and handcrafted features to learned CNN features. ResNet is a residual network architecture and YOLO is an object detection framework; arrows do not imply a universal performance ranking. (**b**) Colony-segment classification accuracy, not whole-plate counting accuracy [33]. (**c**) Study-defined count recovery for Otsu, k-means and U^2^-Net processing with a shared ResNet50 module on validation images [36]. (**d**) Detection mAP@0.5 for YOLOv8n and Colony-YOLO on the same dataset, not direct CFU-counting accuracy [40]. (**e**) Approximate manual and semi-automated counting times from Rodrigues et al. [27]. Panel (**a**) is conceptual; panels (**b**–**e**) replot published results. Comparisons apply within each study; values are not directly comparable across panels. HCF, handcrafted features; CNN, convolutional neural network; SVM, support vector machine.

The persistent shortcomings of manual and rule-based automation have prompted a shift toward learning-based vision systems. CNNs replace handcrafted features with data-driven representations, which allows models to accommodate variation in texture, shape, illumination, and background conditions [37,38]. This flexibility is especially useful in colony analysis, where dense growth, overlap, irregular morphology, and heterogeneous backgrounds routinely confound fixed-threshold approaches [33,35,36,39]. When combined with standardized imaging and total laboratory automation (TLA) infrastructure, deep learning can support repeatable, high-throughput plate reading with less reliance on individual reader judgment. Figure 2a outlines the relationships among selected methods, while panels Figure 2b–e show separate within-study comparisons with explicitly identified outcomes.

### 1.4. Scope and Objectives of This Review

This review examines current methods for AI-enabled colony analysis, with a focus on end-to-end workflows and performance under realistic plate conditions [35,36,39]. Unlike broader reviews of AI-assisted microbial diagnostics, antimicrobial susceptibility testing, or microbial isolation, this review focuses specifically on visible colony-level image analysis and plate-reading failure modes in culture-based microbiology. We first summarize common culture and enumeration formats and the imaging artifacts they introduce, then describe data acquisition, pre-processing, and annotation considerations that shape model behavior. We then examine the major methodological families used in colony analysis, including traditional threshold- and morphology-based pipelines, object detection, semantic segmentation, instance segmentation, and density- or keypoint-based estimation. We also discuss how their strengths and limitations emerge under different plate conditions. Current methods still face limitations in generalization, uncertainty handling, interpretability, and validation under variable laboratory conditions. Uncertainty handling includes routing ambiguous or low-confidence predictions to human review.

Colony-level image analysis also requires microbiological interpretation. Visible colony growth reflects organism-dependent properties such as growth rate, morphology, pigmentation, swarming behavior, filamentous expansion, mixed growth, and interaction with culture media. AI-enabled colony analysis may help standardize culture-based enumeration, improve the reproducibility of colony-level phenotyping, and support the clearer interpretation of microbial growth across research and applied laboratory settings.

The practical meaning of model performance differs across colony-analysis tasks and applications. In clinical microbiology, the main concern may be safe rule-out, reviewability, and turnaround time. In food microbiology, robustness to matrix-derived artifacts and compliance with counting conventions may be more important. In pharmaceutical or environmental monitoring, low-count specificity, traceability, and auditability may matter more than maximizing aggregate accuracy.

This review links recurrent plate-reading failure modes to analytical outputs and validation requirements. It distinguishes evidence for direct enumeration from evidence for plate-level triage and semi-quantitative interpretation, and proposes task-specific reporting and validation criteria. This microbiological focus provides a practical basis for selecting and evaluating methods under different culture and imaging conditions.

## 2. Methodological Overview: From Culture Plates to Digital Data

The performance of AI in colony analysis is closely linked to upstream culture protocols and downstream imaging consistency. Plate type, medium appearance, incubation conditions, and imaging setup all affect the images used to train the model. Standardized acquisition, robust pre-processing, and representative training data are therefore essential if models are expected to generalize beyond a single site or protocol [38,41].

### 2.1. Literature Search Strategy and Scope

This article is a structured narrative review of microbial colony-image analysis. The formal database search was limited to the Web of Science Core Collection. PubMed and Google Scholar were used only to locate individual publications, not for comprehensive database searches. The revision-stage WoS search supplied the 970-record dataset for the descriptive annual distribution in Figure 1b.

The Web of Science Core Collection was searched on 20 September 2026 using the following query: TS = (“colony count*” OR “CFU count*” OR “Colony-Forming Unit count*” OR “enumeration”) AND TS = (“computer vision” OR “digital image” OR “machine learning” OR “deep learning” OR “neural network” OR “artificial intelligence” OR “AI” OR “convolutional neural network” OR “CNN” OR “YOLO” OR “U-Net” OR “segmentation”) AND PY = (1985–2026). The saved search history listed SCI-EXPANDED, CCR-EXPANDED, and IC. No language or document-type restrictions were applied. The full-record export contained 970 unique Web of Science accession numbers (UT), with no duplicate UT records.

The literature discussed in this review was assembled by the first author through the thematic selection of relevant publications and the targeted retrieval of individual sources. It was not confined to the 970-record WoS export. Sources outside that export included relevant colony-analysis and clinical validation studies, methodological and background publications, historical studies, and standards and guidance documents. The thematic scope described below guided the selection of the literature for discussion. Accordingly, Figure 1b describes the search output, not a final included-study set or the complete set of references cited in this review.

The thematic scope includes image-based counting, detection, segmentation, classification, morphology and the growth analysis of cultured bacterial, yeast, and mold colonies or microcolonies. Relevant applications include agar plates, Petrifilm, membrane filtration formats, and automated culture-plate interpretation. Colony-image datasets, synthetic-data methods, and workflow validation are also within scope. Mathematical enumeration, mammalian cell colony assays, tissue imaging, and the counting of individual cells without a cultured microbial colony context are outside the core scope. Studies that use CFU only as an experimental endpoint without examining colony-analysis methods do not provide direct evidence of image analysis performance; culture-method publications may nevertheless inform the background discussion. The preprint and short conference paper are retained as methodological examples; their quantitative performance claims are not reproduced.

Because the studies discussed differ in task definition, culture format, imaging system, organism group, annotation strategy, model output, validation design, and reported metrics, no pooled quantitative comparison or meta-analysis was performed. The narrative synthesis considers intended use, model output, validation design, and external testing.

### 2.2. Common Plating Techniques and Their Imaging Challenges

**Figure 3:**
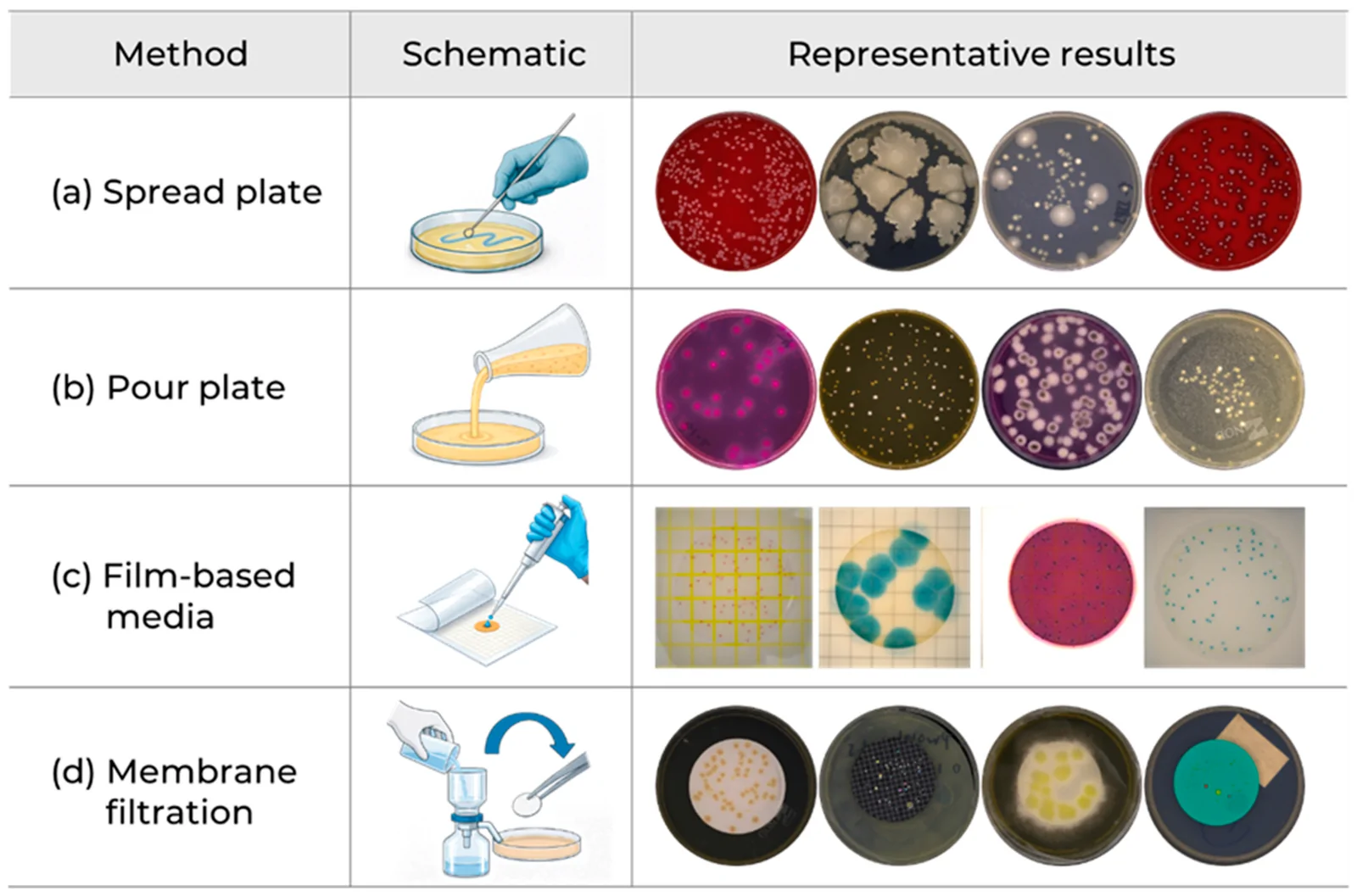
Common plating and culture formats that shape colony-image appearance across sectors: (**a**) direct/spread plating; (**b**) pour plate; (**c**) film-based media such as Petrifilm; (**d**) membrane filtration for low-burden liquid samples. The photographs were taken by the authors, and the schematics were drawn by the authors. No copyrighted material was reproduced, and no permission was required.

Culture formats differ substantially across domains, and each format creates its own image analysis constraints (Table 1) [1,28,42]. As shown in Figure 3, direct or spread plating, pour plating, film-based media such as Petrifilm, and membrane filtration produce different image backgrounds and colony appearances. Spread plating, which is common in clinical urine culture and some pharmaceutical tests, keeps colonies largely on a single focal plane. At high microbial loads, however, it can produce dense overlap and spread phenotypes that obscure discrete colonies [5,26]. Pour plating, which is widely used in food and environmental testing, reduces surface spreading by embedding organisms within agar. Colonies can then form at different depths, which increases variation in focus and contrast, and sample particulates may become trapped and misclassified as colonies [43]. Membrane filtration concentrates low-burden samples onto a filter, but grid lines and membrane coloration introduce structured background signals. Colonies are often small and crowded, which increases demands on resolution and segmentation [9,16]. Film-based culture formats such as Petrifilm should be treated as distinct analytical platforms rather than simple variants of conventional agar plates. Their grid patterns, chromogenic reactions, gas bubbles, pinpoint colonies, and adherent growth differ materially from standard Petri-dish media and often require method-specific handling [44,45,46].

Cross-format transfer is difficult because both image appearance and counting conventions change across culture formats. Agar plates, pour plates, Petrifilm, and membrane filters differ in background texture, contrast, focal depth, grid patterns, chromogenic reactions, bubbles, debris, and colony density. They also differ in how edge colonies, merged colonies, pinpoint colonies, artifacts, and TNTC regions are counted or excluded. TNTC (too numerous to count) describes plates whose colony density exceeds the upper counting limit of the method. Under standard counting conventions (e.g., ISO 4833-2:2013 [47] and BAM Chapter 3 [1]), such plates are not assigned a precise count; the correct action is to report an estimated count, invalidate the plate, or dilute and re-plate the sample. Models trained on one format may therefore learn format-specific visual cues and counting rules, so direct transfer to another format requires validation and, in many cases, retraining.

**Table 1:** Quantitative comparison of common culture methods and implications for AI analysis.

Method	Typical Inoculation Volume	Method-Specific Counting Criteria	Typical Media	Application Fields	Key Challenges for AI Analysis	References
Spread plate	0.1 mL on a 90 mm plate; urine streaking uses calibrated loops (1 or 10 µL)	USP <61>: fewer than 250 colonies for TAMC and fewer than 50 for TYMC; criteria depend on the method	Blood agar, MacConkey agar, Tryptone Soya agar, Nutrient agar, Chromogenic agar, Sabouraud Dextrose agar, etc.	Clinical: Urine culture. Pharmaceutical: Non-sterile product testing	Overlap; mixed morphotypes; pigments; spreading growth may invalidate enumeration	[11,27,32,42,48]
Pour plate	1.0 mL	BAM Chapter 3 (May 2025): 15–300 colonies per plate	Plate-Count Agar, Potato Dextrose Agar, Reasoner’s 2 agar(R2A), etc.	Food: enumeration of indicator bacteria and specific pathogens; Environmental: Water quality testing.	Colonies at different focal depths; embedded debris; subsurface colonies	[1,10,42,49]
Membrane filtration	1–100 mL, method-dependent filtered volume	Method- and organism-specific; no universal range	Nutrient agar, R2A agar, Sabouraud Dextrose Agar, etc.	Water for monitoring Pharmaceutical products: soluble drugs, microbiological limit test	Grid or background; low contrast; small colonies; edge convention; low-count artifacts	[1,15,16,17,18,50]
Film-based media	1.0 mL	Petrifilm YM: 15–150 colonies per plate	Ready-to-use film containing method-specific nutrients, gelling agents and indicators	Food: Widely used for total aerobic count, *E. coli*, Coliforms, and yeast/mold in various food products.	Printed grid; chromogenic color; gas bubbles; spreading or adherent growth	[45,51,52]

Note: The method-specific criteria refer to plate selection for enumeration; they are not universal ranges shared across culture formats. Regulatory or product-release limits, such as ≤10 CFU/100 mL for certain water-monitoring contexts, should not be interpreted as analytical counting ranges; they represent compliance or acceptance criteria rather than the counting capacity of the method. The integer count obtained from an individual plate is an intermediate observation rather than, in itself, the final microbiological result. Under standard plate-count procedures, counts from eligible replicate plates and, where applicable, successive dilutions are combined with the inoculated volume and dilution factor to estimate the concentration in the original sample, commonly expressed as CFU/mL or CFU/g [53]. TAMC, total aerobic microbial count; TYMC, total yeast and mold count; CFU, colony-forming unit. BAM APC: Bacteriological Analytical Manual Chapter 3: Aerobic Plate Count.

### 2.3. Data Acquisition and Pre-Processing: The Need for Standardization

Deep learning models are susceptible to domain shift. Changes in imaging hardware, lighting, incubation timing, or media appearance between training and deployment can degrade performance [38]. Standardized image acquisition is therefore a practical prerequisite for reliable AI plate reading [32]. TLA systems such as BD Kiestra and Copan WASPLab provide controlled imaging conditions, including consistent geometry, timing, and multi-modal illumination, which reduce variability and support downstream automated interpretation [32,37,39,54]. In practice, standardized acquisition and consistent pre-processing help models generalize to routine use [32,41].

### 2.4. The Role of Data Augmentation and Synthetic Data

Data availability continues to limit model development, especially when expert annotation is required [38,55]. Colony labeling can be time-consuming and error-prone, and dense or confluent plates introduce ambiguity in defining a countable unit. Two complementary strategies are commonly used to address the scarcity of labeled data [56]. First, data augmentation expands the effective training set through plausible geometric transformations such as rotation, scaling, flipping, and translation, together with photometric adjustments and noise perturbations [40,57]. These operations approximate real imaging variation and help models tolerate these changes. For example, Zhang et al. used an object-covering augmentation approach to expand five initial images into 960 augmented images (864 training images and 96 validation images), which improved performance while reducing the manual annotation burden by about 80% [57].

Synthetic-data generation provides another way to expand training datasets, especially for rare phenotypes or difficult scenarios. Generative adversarial networks and related models can learn the joint appearance of colonies and agar backgrounds, which enables realistic image synthesis or style transfer [56,58]. Pawlowski et al. generated synthetic training images from 100 real images and evaluated the resulting detector on real AGAR test images [56]. The reported counting error was close to that of a detector trained on a much larger real dataset, while detection mAP was lower; these are distinct outcomes [56]. Synthetic datasets do not remove the need for representative real-world validation, but they can be useful for stress-testing models on extreme overlap, unusual morphologies, or specific artifact patterns that are underrepresented in routine collections [59]. Limited-image and synthetic-data strategies can reduce annotation cost, but they may overfit to a narrow range of media, colony morphologies, imaging conditions, and artifacts. Synthetic data may improve detection sensitivity by increasing colony-like examples, but any gains should be verified on independent real-image test sets, including specificity and false-positive behavior for bubbles, scratches, debris, and plate-edge effects.

### 2.5. Publicly Available Datasets and Benchmarking Constraints

Open datasets are essential for reproducible benchmarking, but available colony-image resources remain limited in scale, organism diversity, culture format, annotation type, and deployment realism. Table 2 summarizes representative public or reusable resources that are directly relevant to colony detection, segmentation, counting, or synthetic-data generation. These datasets are valuable for method development, but they do not eliminate the need for external validation on the media, organism groups, imaging systems, and counting rules used in the target laboratory workflow. Manual colony counting remains the de facto reference standard, but it is itself uncertain because inter-reader agreement is rarely reported and reader performance varies with experience, fatigue, and viewing conditions. Where studies use manual counts as the reference, the interpretation of model error depends on how countable units, reader disagreement, and adjudication are handled. Future benchmarking should use an adjudicated multi-reader reference. Disagreement between an AI result and a human count does not necessarily indicate an AI error; uncertain cases require the review and adjudication of the reference count.

## 3. Core AI Architectures for Colony Enumeration

**Figure 4:**
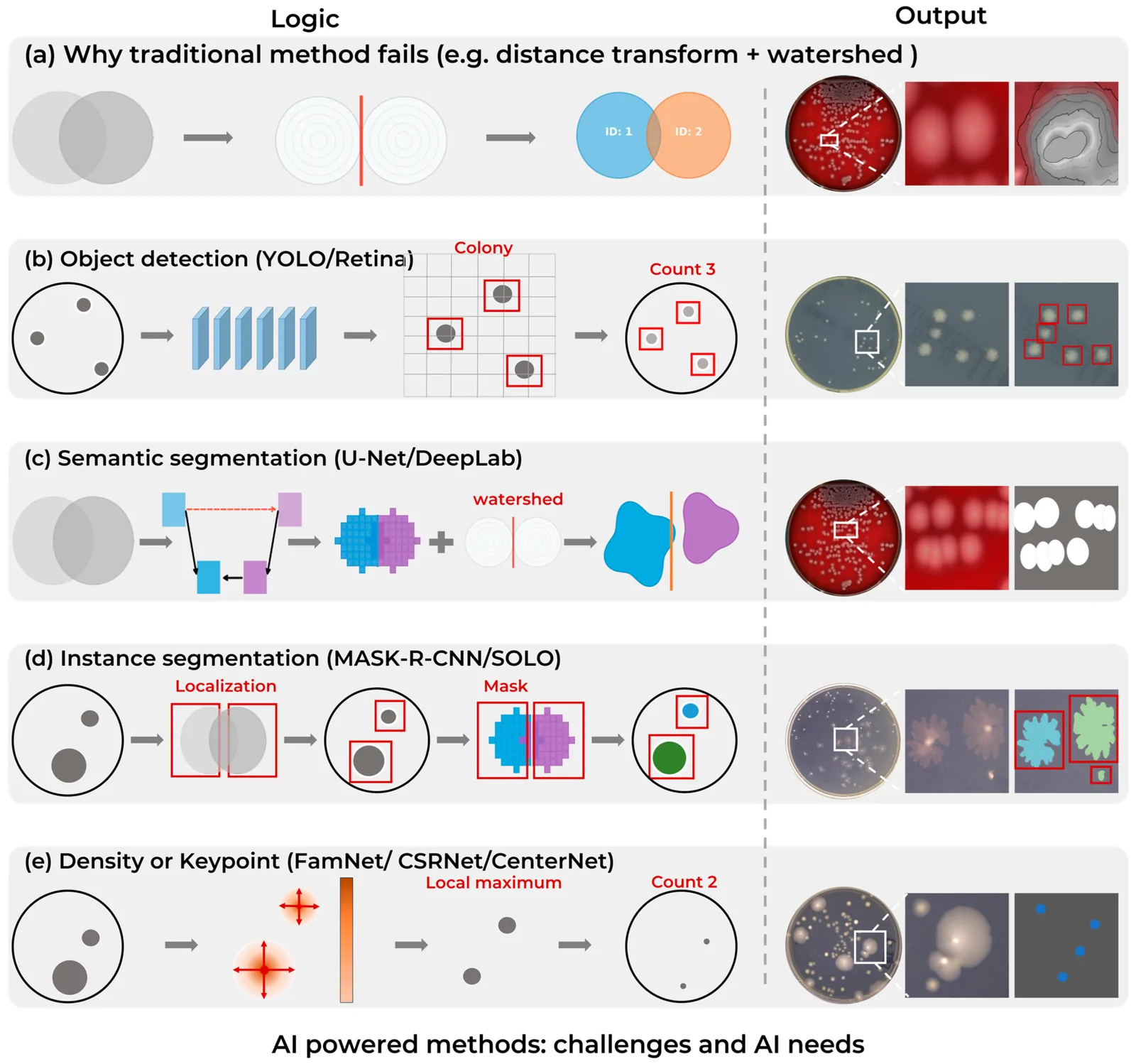
Representative colony-analysis paradigms and their outputs. From top to bottom: traditional threshold/morphology methods (**a**), object detection (**b**), semantic segmentation (**c**), instance segmentation (**d**), and density/keypoint counting (**e**). Left: computational logic. Right: reviewable output. The authors generated Figure 4 based on their own dataset.

**Figure 5:**
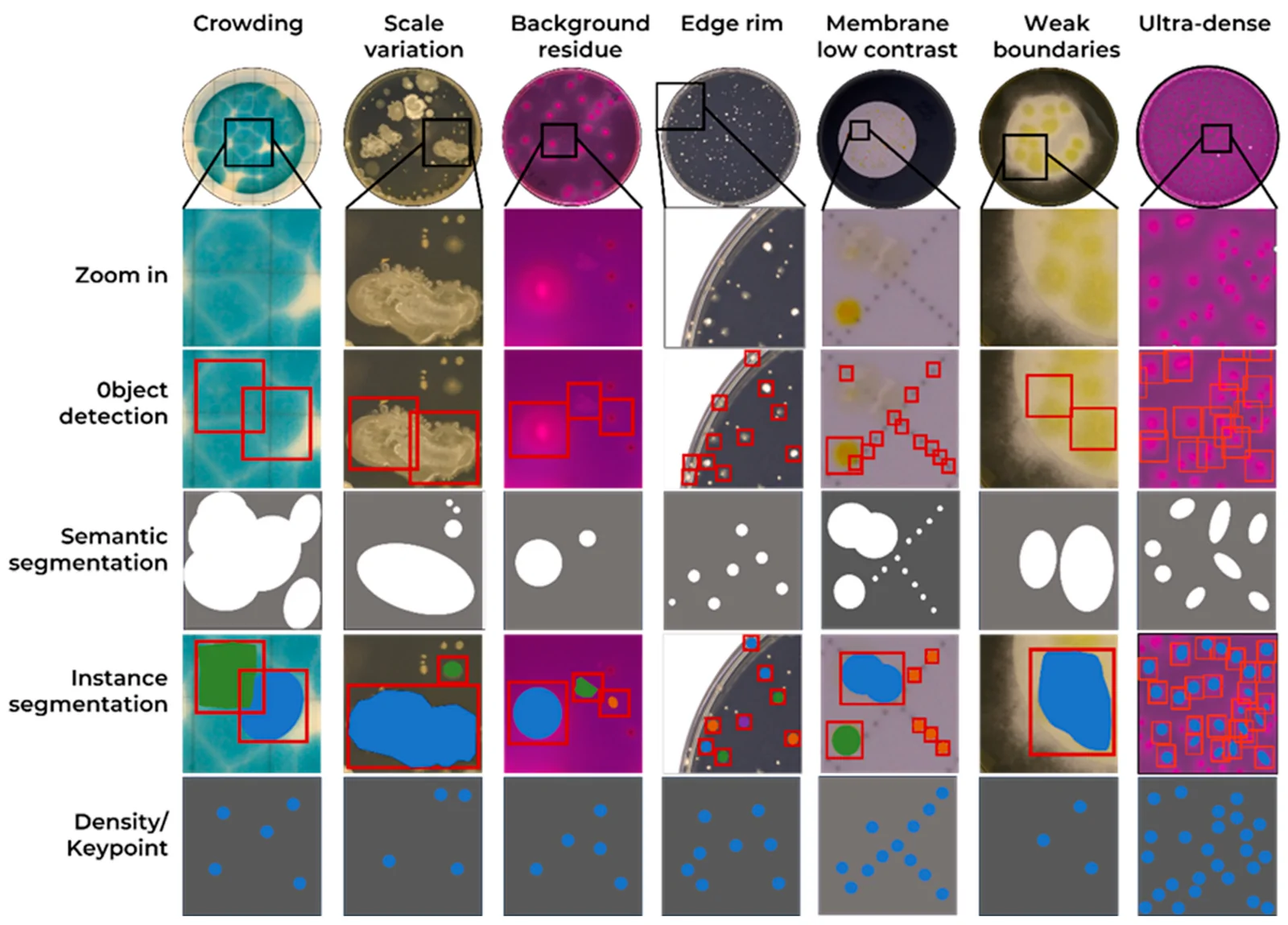
Representative failure scenarios that shape method choice. Columns show crowding/occlusion, scale variation, background residue, plate-rim partial targets, membrane/filter low contrast, weak internal boundaries, and ultra-dense/TNTC growth. The authors generated Figure 5 based on their own dataset.

**Figure 6:**
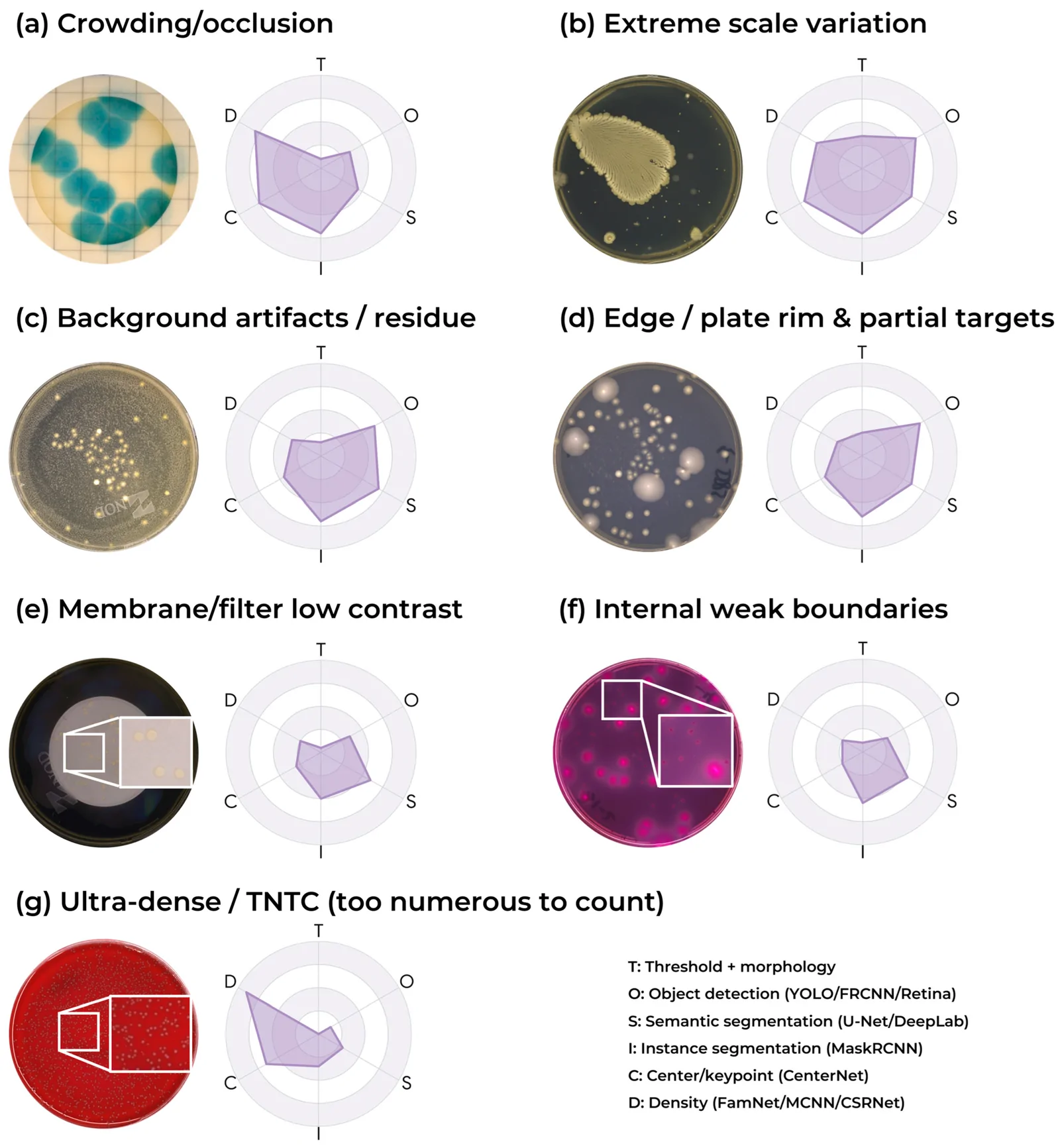
Conceptual mapping between common colony-analysis scenarios and model families that may be better aligned with their dominant technical demands. Outward extension indicates the authors’ qualitative emphasis, informed by methodological reasoning and the literature discussed in Section 3.6 [36,40,63,64,65]. These hypotheses are not derived from a common benchmark or calibrated scoring system; radial positions and polygon areas have no validated quantitative meaning. Scenarios may coexist, and workflows may combine methods. In panel (**g**), density estimation denotes exploratory image analysis, not validated CFU or biomass estimation. T, threshold/morphology; O, object detection; S, semantic segmentation; I, instance segmentation; C, center/keypoint counting; D, density estimation. Author-supplied photographs are illustrative, not test images from the cited studies.

Figure 2, Figure 3, Figure 4, Figure 5 and Figure 6 describe colony-analysis methods, culture formats, and practical challenges. Figure 2 presents source-specific findings, whereas Figure 3 illustrates culture formats that shape image appearance. Figure 4 organizes the principal methodological families by their computational logic and output format. Figure 5 illustrates practical visual challenges that may affect colony analysis. Figure 6 presents an author-proposed conceptual overview of potential matches between tasks and methods across these scenarios. Its profiles express qualitative judgments about candidate approaches rather than measured differences in performance. The rationale for these judgments and their limitations are discussed in Section 3.6.

### 3.1. Thresholding and Classical Computer Vision

Before deep learning, automated colony counting relied mainly on rule-based image-processing pipelines [25,26]. Typical workflows combine denoising and background correction with global or adaptive thresholding to separate colonies from agar. They then use contour analysis, morphology, distance transforms, or watershed splitting to estimate colony number [25,63,66]. The output is usually a binary mask, a set of contours, or a final integer count with limited information about individual colonies. These methods remain attractive when annotation resources are limited and the imaging setup is highly standardized. Performance can deteriorate when imaging conditions differ from those used for calibration. Overlap and crowding can merge colonies into a single component. Residue and uneven backgrounds can create false positives. Weak internal boundaries can prevent watershed splitting, and edge effects or membrane grids can distort contour-based rules [26,27,67,68].

### 3.2. Object Detection Approaches (e.g., YOLO)

Object detection treats colony counting as an instance-localization task. Each colony is represented by a predicted bounding box, class label, and confidence score [57]. The output is compact and easy to review, which makes this approach attractive for high-throughput workflows in which a human can quickly confirm or correct candidate colonies rather than inspect every pixel. YOLO-style one-stage detectors are useful when processing speed and deployment efficiency are priorities [40].

In practice, object detectors perform well when colonies are discrete, reasonably separated, and visually salient. YOLO variants have been evaluated using detection metrics [40,57,69]. Mean average precision (mAP) summarizes precision–recall performance under specified bounding box overlap criteria; it does not directly measure the agreement between predicted and reference colony counts. Detection can also remain robust under moderate overlap or mixed morphology when object identity is still visually recoverable. The box-based output becomes less informative, however, when colonies are highly confluent, extremely small, partially clipped by the plate rim, or embedded in structured backgrounds such as membrane grids or debris-rich food matrices [36,40,69]. Because a box does not explicitly model colony contour, object detection often needs downstream rules when laboratories need to assess exact colony extent and phenotype shape, or whether a faint border object should be counted.

### 3.3. Semantic Segmentation Approaches (e.g., U-Net)

Semantic segmentation, a pixel-level classification task, assigns a class label to each pixel, for example, colony versus background or multiple phenotype classes, and produces a dense mask rather than a sparse list of boxes [33,36]. This output is especially useful when colony shape, area, and boundary continuity carry biological meaning, or when the plate contains irregular, elongated, or partly merged colonies for which a rectangular box is a poor description. In microbiological terms, segmentation can delineate interpretable growth regions such as individual colony boundaries, confluent zones, swarming fronts, filamentous margins, hemolytic areas, or chromogenic colony regions. However, delineating a swarming front does not make the underlying plate enumerable, because discrete colony-forming units can no longer be reliably recovered from confluent swarming growth.

Encoder–decoder architectures such as U-Net and DeepLab remain influential because they combine global context with fine localization through skip connections and multi-scale features [33,70]. Representative studies show that segmentation can support both counting and downstream interpretation. For example, Cao et al. combined U2-Net with ResNet50 for colony segmentation and counting, reporting an overall colony-counting recovery of 97.82% under the study’s definition [36]. For positive reference counts, recovery was defined as one minus the absolute relative count error; zero-colony-image crops were evaluated using recall for the zero-colony class. This summary does not establish the direction of bias or per-plate limits of agreement. Segmentation provides pixel-level information when colony shape or local overlap matters, but successful enumeration still depends on separating adjacent colonies at weak boundaries. A related fully automated deep learning pipeline for high-throughput colony segmentation and classification has also been reported [71]. Although developed for a specific yeast genetic-screening assay rather than routine colony enumeration, this study illustrates how segmentation and classification can be integrated into an end-to-end colony-analysis workflow under well-defined imaging conditions.

This reliance on boundary quality creates characteristic failure modes. When colonies fuse into a continuous region, semantic segmentation can under-separate touching objects unless auxiliary boundary labels, distance transforms, or watershed post-processing are added [33,36]. Low-contrast filters, weak halo boundaries, and severe background residue can also degrade pixel classification. Segmentation is most informative when colony or growth-region boundaries remain biologically interpretable, for example, for touching but still distinguishable colonies, hemolytic areas, chromogenic regions, or morphology-based phenotyping. It should not be presented as a means of recovering an exact colony count from confluent or swarming growth. For plates showing unrestricted *Proteus* spp. swarming, the appropriate automated output is a non-enumerable or review-required flag rather than a numerical count [72,73].

### 3.4. Instance Segmentation Approaches (e.g., Mask R-CNN, SOLO)

Instance segmentation localizes individual colonies and assigns each one a separate mask. It outputs a bounding box, class label, confidence score, and an individual binary mask for each colony. This representation preserves object identity while also recovering shape, which makes it particularly useful when neighboring colonies touch but remain separable. Compared to semantic segmentation, instance segmentation usually requires richer annotations. In return, it produces outputs that are easier to audit, edit, and connect to downstream morphology analysis.

Naets et al. described a Mask R-CNN workflow for distinguishing two bacterial-colony classes on Petri dishes, with an interface for laboratory staff to review and correct predictions [64]. This arXiv preprint is retained as an example of an interactive workflow, not as evidence of validated counting performance; its numerical performance results are not reproduced here. Instance masks can support the review of touching colonies and mixed morphology when individual objects remain distinguishable. Confluent growth, weak boundaries, and small artifacts still require explicit counting and review rules.

### 3.5. Density/Keypoint-Based Counting Approaches (e.g., FamNet, CSRNet, CenterNet)

Density-based methods integrate a predicted density map to estimate a count, whereas keypoint-based methods localize colony centers. Both can avoid the need for full contour annotations, but they provide less information about individual colony boundaries. Zheng et al. described a few-shot density-estimation approach using user-selected colony exemplars in a short conference paper [74]. This source is retained to illustrate the method, without reproducing its reported MAE or using it to establish counting accuracy, agreement, or superiority over other approaches.

These methods warrant investigation when box or mask annotation is difficult. Their use on crowded images does not make confluent or otherwise non-enumerable plates suitable for exact CFU reporting. The trade-off is that boundary fidelity is lost, phenotype review is more difficult, and performance can be sensitive to shifts in background texture, plate edges, or colony size [74]. Complementary unsupervised or multi-modal strategies, including autoencoder-based representation learning and hyperspectral imaging with lightweight classifiers, remain useful when labels are scarce or when debris rejection matters more than contour recovery [43,75]. CSRNet is a density-estimation method, whereas CenterNet is a keypoint detector; both produce count-oriented outputs rather than masks.

Transformer-based detectors and segmenters (DETR, Mask2Former, SegFormer, Swin), promptable segmentation models (SAM/SAM2), and self-supervised pretraining are emerging general-purpose methods. Their applicability to routine colony enumeration requires task- and setting-specific validation. Table 3 provides a non-comparative summary of representative AI studies relevant to colony analysis and enumeration, grouped by evidence category.

### 3.6. Task-Specific Method Selection

Method selection should follow the required output and dominant failure mode, rather than an assumed ranking of architectures. Complementary functions have been combined in published workflows: U^2^-Net segmentation with ResNet50 aggregate counting [36], and multi-loss U-Net with plate-region and reflection handling [65]. However, additional processing worsened some AGAR results [65]. Residual errors involving small or adherent colonies in Colony-YOLO [40], the proposed review interface in a Mask R-CNN preprint [64], and classical bubble and dish-edge handling in OpenCFU [63] show why the complete workflow needs evaluation.

Figure 6 presents literature-informed conceptual hypotheses, not validated performance rankings; its radial positions are not established by these studies. Proposed center/keypoint and density-based applications remain methodological extrapolations with limited direct validation for the illustrated scenarios. Hybrid methods require comparison with strong single-model baselines using common data, explicit reference-counting procedures, component ablation, and workflow costs. Results on ordinary agar do not establish performance on low-contrast membranes, and confluent or otherwise non-enumerable growth should not be assigned an unsupported exact CFU count.

## 4. Applications and Performance in Key Sectors

Table 4 summarizes industry-specific challenges in microbial enumeration and the targeted AI solutions reported in the literature. Across sectors, AI colony analysis is moving from proof-of-concept studies toward operational tools, often within automated laboratory workflows [39,54]. Figure 5 highlights recurring challenge patterns such as crowding, scale variation, residue, plate-rim effects, membrane low contrast, weak internal boundaries, and TNTC growth. Reported performance therefore depends not only on algorithm family, but also on task definition, culture format, sample matrix, and the surrounding laboratory workflow [76]. Throughout this review, performance metrics are reported together with their validated output type (triage, semi-quantitative, detection, counting, or segmentation), as summarized in Table 3.

### 4.1. Clinical Microbiology: Stronger Evidence for Plate-Level Triage than for Direct Colony Counting

Clinical microbiology has relatively well-developed evidence from laboratory workflows for AI-enabled culture-plate interpretation, particularly for urine-culture screening and plate-level triage [5,39,76]. In this setting, the primary output is usually a plate-level category, automated rule-out decision, or review flag rather than a colony-by-colony count. APAS, WASPLab, BacterioSight, and DeepColony therefore support clinical workflow interpretation, but their reported sensitivity, specificity, area under the receiver operating characteristic curve (AUC), or accuracy should not be interpreted as direct CFU-counting performance.

Published urine-culture studies illustrate this distinction. WASPLab growth detection reported 99.8% sensitivity and 72.0% specificity in 12,931 urine samples [76]. A multicenter APAS evaluation of 9224 urine specimens reported 99.0% sensitivity and 84.5% specificity for growth detection on blood agar [5]. BacterioSight evaluated 114,074 urine-culture images and reported >97% plate-classification accuracy, with AUC values of at least 0.98 within institutions and at least 0.90 across institutions [39]. Dedicated APAS evaluations reported discordant classifications relative to manual reading that required adjudication, and automated counts did not always agree with the reference standard [6,7]. These metrics reflect plate-level triage or semi-quantitative performance and do not establish CFU-counting accuracy.

These metrics are appropriate for triage, safe rule-out, and review prioritization, but direct enumeration requires CFU counts, colony centers, bounding boxes, or instance masks evaluated against manual or adjudicated reference counts [33,36,40,57]. Relevant enumeration metrics include mean absolute error (MAE), bias, agreement with reference counts, density-stratified error, and performance on small, overlapping, or low-contrast colonies.

Regarding operational outcomes, Faron et al. reported a mean-time-to-result of 18 h 44 min for software-analyzed negative urine cultures in the CPSE/WASPLab workflow, compared to 24 h 18 min for conventional culture [77]. However, software completion times did not include additional identification procedures required for some positive cultures, and the study did not quantify reductions in staffing requirements or net costs [77]. Auto-release criteria and human-review requirements should be established through local workflow validation.

Clinical plates also contain morphologies that complicate both manual reading and automation, including crowded growth, mixed morphotypes, plate-rim ambiguity, swarming *Proteus* spp., and spreading or pigment-producing *P. aeruginosa* colony appearances [19,72,73]. Morphology-aware AI and chromogenic-media workflows can help flag clinically relevant subpopulations or targeted pathogens, but these outputs should still be interpreted within their specific validation setting [35,77,78]. AI-assisted plate triage has been reported to reduce manual-review burden and shorten reporting time in selected workflows, particularly for negative or clearly unremarkable cultures [28,94,95]. Color development on chromogenic media may vary with the medium formulation, incubation time and temperature, inoculum or colony density, and strain phenotype. For automated imaging, illumination, exposure, and white-balance settings must therefore be standardized; color-based assignments should be reported as presumptive identification and confirmed according to the medium-specific instructions and laboratory SOP.

### 4.2. Food Industry

In food microbiology, colony counting is tightly standardized and usually requires accurate absolute enumeration under regulatory frameworks such as ISO methods and FDA-aligned expectations. This applies to indicator organisms and pathogens such as *E. coli*, *Salmonella* spp., and *Listeria* spp. [1,12,80,96,97,98]. Food samples can also present difficult visual conditions, as summarized in Figure 5, including debris-rich backgrounds, extreme colony-size variation, gas bubbles, color-changing indicators, and plates that become TNTC at high burdens. Food-sector AI systems therefore need to be robust not only to biological variation, but also to matrix-derived artifacts and method-specific counting rules.

Matrix interference is a major challenge because food particulates embedded in or on the agar can resemble colonies in color, size, or shape, especially in pour plates [43,98]. Hyperspectral reflectance imaging offers one way to distinguish colony growth from food debris using wavelength-dependent differences in surface reflectance. Using such features, Shi et al. reported a coefficient of determination of R^2^ = 0.9998 between model-predicted and manually determined colony counts [43]. This association does not by itself establish agreement or interchangeability with reference counts.

Food and pharmaceutical testing can involve very small or low-contrast colonies that are difficult to detect consistently. At the other extreme, confluent or TNTC growth prevents reliable separation of countable objects. Density estimation may support exploratory image analysis in such cases, but it does not make an invalid plate suitable for exact CFU reporting.

Film-based media such as Petrifilm create distinct image analysis requirements because printed grids, chromogenic reactions, gas bubbles, microcolonies, and adherent growth patterns differ from standard agar plates [44,46,51,52]. Direct AI evidence for Petrifilm-specific image interpretation remains limited; therefore, these studies are used here as format-specific background rather than as direct AI-performance evidence. Future AI applications on Petrifilm formats will likely require format-specific training and explicit artifact handling rather than direct transfer from standard agar datasets.

Operational benefits should be evaluated using measured image analysis time and hands-on review time across colony-density strata, including any time required for manual correction. A conference report describes medium-specific YOLO models for food-industry colony detection [69]. Beyond speed, digital image archiving also improves traceability and supports more consistent application of counting rules across shifts and sites [54,79].

### 4.3. Pharmaceutical Quality Control

Pharmaceutical quality control and environmental monitoring operate under strict regulatory expectations, including United States Pharmacopeia (USP) and European Pharmacopeia (EP) methods and environmental guidelines, and often require high sensitivity for low-level contamination [9,11,81,99]. Depending on the product, workflows include sterility testing or microbial limits testing with acceptance criteria spanning total aerobic microbial count, yeast and mold count, and specified organisms [48,99,100]. In these settings, the main analytical challenges often differ from those in clinical or food microbiology. Low-count specificity, membrane or filter low contrast, plate-rim exclusions, and the need to distinguish true colonies from scratches, bubbles, fibers, or media defects become particularly important. Direct, externally validated evidence for AI-based colony enumeration in pharmaceutical QC remains limited, particularly for low-count and artifact-rich plates.

Low-bioburden applications require both sensitivity and specificity. In Grade A environmental monitoring, EU GMP Annex 1 specifies no growth, and any detected growth should trigger an investigation [90]. False positives from scratches, bubbles or media defects may therefore prompt unnecessary investigations, whereas missed colonies may conceal contamination. For this application, validation should assess per-plate false-positive rates at a reference count of zero and false-negative rates at a reference count of one. Rule-based counters are especially vulnerable because they rely on fixed thresholds for size and contrast. Segmentation- and instance-based AI models can be trained to distinguish artifacts from colonies under sparse growth, but they still require site-specific validation to show that increased sensitivity does not simply create more false alarms.

Deployment in regulated environments requires validation designs that demonstrate accuracy, precision, linearity, and robustness relative to reference methods [8,81]. AI systems that log decisions and archive images can strengthen traceability and support data-integrity expectations by providing an audit trail for inspections and quality assurance [32,54]. Pharmaceutical and environmental-monitoring workflows are often conducted under predefined media, incubation, and imaging protocols. Such standardization can reduce avoidable input variability, but site- and protocol-specific verification remains essential [85,86,87].

### 4.4. Environmental Monitoring

Environmental monitoring in pharmaceutical cleanrooms generates large numbers of plates requiring consistent reading and documentation. Peer-reviewed reports describe the evaluation, qualification, and validation of automated incubation, advanced imaging, and colony-counting systems for pharmaceutical applications [85,86,87]. APAS-specific environmental-monitoring reports include a conference poster and a short published report [88,89]. These sources are considered preliminary, workflow-specific evidence and do not establish broadly generalizable AI counting performance.

Environmental monitoring of water and air often relies on membrane filtration to concentrate low-burden samples onto a filter surface [9,15,16,17,91,92,93]. The membrane background color and grid pattern introduce structured noise, and the resulting colonies are frequently small and crowded, which increases demands on imaging resolution and segmentation performance. These conditions combine several sources of error illustrated in Figure 5: membrane low contrast, edge effects, and crowding. Automated membrane-filter plate reading could therefore assist the routine monitoring of critical systems such as hospital water supplies, especially when combined with standardized acquisition and image archiving that supports audit. However, direct AI validation for membrane-filter formats remains limited and requires further study.

Quantifying fungi and molds presents additional challenges because filamentous growth is irregular, diffuse, and often cannot be separated into discrete colonies. CNN-based models can move beyond simple shape rules by learning textural signatures associated with fungal morphology. Recent work suggests that deep learning can classify fungal genera from macroscopic plate images with high accuracy [62,83]. For example, using the OpenFungi dataset of macroscopic and microscopic images, a CNN achieved 99.79% accuracy on macroscopic images and 97.82% on microscopic images [62]. These datasets focus on recognition and classification rather than direct enumeration, but they still provide useful benchmarks for morphology-aware analysis. Such morphology analysis may inform future methods for counting or segmenting complex filamentous mats. Ongoing challenges include limited dataset size, class imbalance, and substantial morphological plasticity, which make augmentation, resampling, and architecture selection especially important [62,82]. For filamentous fungi, CFU is an operational rather than a direct organism count because a colony may arise from a spore or hyphal fragment, and plates obscured by rapidly spreading mold growth may be non-enumerable under the applicable USP or ISO method.

## 5. Metric Comparability and Operational Impact

Performance metrics should be selected according to the intended output rather than treated as interchangeable measures of “accuracy” (Table 5). Counting studies should report absolute and signed errors across pre-specified density strata and assess agreement against an adjudicated reference using Bland–Altman limits of agreement and the concordance correlation coefficient (CCC), rather than relying on correlation or R^2^ alone. Classification accuracy measures agreement of labels, and detection mAP measures localization and classification under specified overlap criteria. Neither establishes the agreement of colony counts. MAE describes the magnitude of count error but does not identify systematic overcounting or undercounting. Count recovery must be interpreted using the source definition and the distribution of individual errors; a high summary value alone is insufficient to establish interchangeability. Acceptance limits should be specified for the intended microbiological use. Detection studies should report AP across multiple intersection-over-union thresholds and analyze performance by colony size, whereas segmentation studies should include overlap- and instance-aware metrics that expose merged or fragmented predictions. Triage systems require sensitivity and specificity with confidence intervals at a pre-specified operating point, together with predictive values and the resulting manual-review burden. Validation should reflect the intended use, include external-site testing where applicable, lock the model before testing, and explicitly examine low-count, non-enumerable/TNTC, and artifact-rich plates. A validation report should identify the test sites, imagers, media and organism groups, justify sample size, and separate independent plates or batches from repeated images. Challenge testing should include bubbles, scratches, fibers, condensation, rim effects and food debris. Deployment requires documented drift monitoring and triggers for revalidation after changes to the model or workflow (Table 6).

Sampling variability must also be distinguished from image-reading error. Under an ideal Poisson model, replicate plate counts with expected count n have a relative standard deviation of $1/\sqrt{n}$; at *n* = 25, this is 20% [101]. This concerns the variability between sampled aliquots and plates, not an irreducible error in the automated versus reference reading of the same plate image.

**Table 6:** Sector-specific validation and regulatory considerations for automated colony-analysis systems.

Sector	Instrument or Use	Principal Framework
Pharmaceutical quality control	Automated colony counter or alternative/rapid microbiological method	USP <61>, <62>, <71>, <1111>, <1223>; Ph. Eur. 5.1.6; PDA TR33
Pharmaceutical environmental monitoring and water	Automated counter or AI plate readers	EU GMP Annex 1; USP <1231>; 21 CFR Part 11; EU GMP Annex 11; GAMP 5
Food microbiology	Automated colony counters; film-plate readers	ISO 16140-2:2016 [102]; ISO 16140-3:2021 [103]; ISO 11133:2014 [104]; ISO 19036:2019 [105]
Clinical microbiology	Digital plate reader or AI-assisted triage system	ISO 15189:2022 [106]

Abbreviations: USP, United States Pharmacopeia; Ph. Eur., European Pharmacopeia; PDA, Parenteral Drug Association; TR, Technical Report; EU, European Union; GMP, Good Manufacturing Practice; CFR, Code of Federal Regulations; GAMP, Good Automated Manufacturing Practice; ISO, International Organization for Standardization. Numbers in angle brackets denote USP general chapters; PDA TR 33 denotes Technical Report No. 33; 21 CFR Part 11 denotes Title 21, Part 11 of the Code of Federal Regulations.

Figure 2 separates methodological relationships from quantitative comparisons. Panel a is conceptual: ResNet is a residual CNN architecture and YOLO is a detection framework, so neither label alone establishes superiority for colony counting. Panel b shows the within-study colony-segment classification comparison of Ferrari et al. [33]. Panel c compares Otsu thresholding, k-means clustering and U^2^-Net segmentation with a shared ResNet50 counting module [36]. It evaluates alternative processing pipelines, not independent standalone classifiers. Panel d compares two detectors on the same dataset using mAP@0.5 [40]. These results support specific advantages within the reported experiments, but do not establish a cross-study ranking of counting accuracy.

Operational benefit likewise depends on the tested workflow. In Figure 2e, Rodrigues et al. reported approximate manual counting times of 20–162 s and algorithm times of 36–44 s for plates containing 30–300 colonies [27]. The semi-automated method was slower at the two lowest plotted counts and faster from 100 colonies upward in that experiment. These timings describe a particular image analysis implementation and hardware setting, not a general AI speed advantage or end-to-end laboratory turnaround time. Hands-on correction, review, acquisition and documentation should be included when evaluating deployment efficiency.

## 6. Current Challenges and Limitations

Several barriers continue to limit the routine deployment of AI-assisted colony analysis, including limited external validation, inconsistent annotation and reference standards, insufficiently transparent model decisions, and the absence of widely adopted reporting and revalidation frameworks [38,41].

Technical and data constraints contribute to these limitations. Deep learning models often provide little explanation for a plate classification or for a count that differs from human judgment. This remains a major barrier to trust in clinical and regulated contexts [38,41,107]. Performance also depends on training-data quality and coverage [38,55]. Small, unrepresentative, or inconsistently annotated datasets can produce models that fail under new media, species, or morphologies such as confluence and filamentous growth. Data augmentation and synthetic data can help, but they do not replace the need for representative multi-site datasets and clearly defined reference standards [56,57,82]. Dataset bias may arise from the over-representation of particular organism morphologies or imaging conditions and from inconsistent annotation rules, such as whether touching colonies are labeled separately; these issues can compound laboratory-to-laboratory differences in cameras, illumination, media and incubation time [39,56]. Related challenges in data representativeness, generalization and transparency are discussed in agriculture, where AI-based computer vision is increasingly integrated with Internet of Things (IoT) sensing and explainable AI [108].

Human review does not guarantee that errors will be detected. Automation bias and complacency may cause reviewers to miss AI errors, and deskilling may occur when AI auto-releases easy plates. A distinction should be made between AI-assisted release, where a human authorizes the result, and AI-autonomous release, where no human reviews it. Under ISO 15189:2022, responsibility for a released count remains with the laboratory, not the vendor [106]. LIS flagging and audit trails should record the image, model ID and version, confidence, reviewer ID, and timestamp. The ability of human reviewers to detect and correct AI errors should therefore be evaluated in the intended colony-analysis workflow rather than assumed.

The review relies on one documented database search and the selective retrieval of additional sources. The bibliography was not derived from a completed eligibility assessment of all retrieved records, which limits the completeness and reproducibility of literature selection and may introduce selection bias.

Foundation models, including promptable segmentation and vision–language models, may support future colony analysis, but they remain emerging tools that require task-specific validation. Algorithmic performance alone does not establish readiness for regulated use. Such deployment will require validated performance under the intended use conditions, transparent error handling, calibrated confidence reporting, documented model updates, and regulatory alignment appropriate to the clinical, pharmaceutical, food, or environmental context.

Standardization and regulatory requirements also affect deployment. The field still lacks widely adopted validation protocols and performance-reporting conventions tailored to colony analysis. This makes cross-study comparison difficult and slows acceptance in high-stakes settings [81,109,110]. AI-integrated automation platforms also require capital investment and staff training, which may be difficult for smaller laboratories [32,54]. Wider adoption will depend on transparent benchmarking, clearer regulatory pathways, and deployment practices that include drift monitoring, periodic revalidation, and documentation suitable for audit.

Classical methods remain relevant when annotation resources are limited and imaging conditions are controlled; performance should be assessed for the specific implementation and task [63]. Section 3.6 discusses the evidence and limitations of combining complementary analytical functions.

## 7. Conclusions and Future Outlook

Culture-based colony enumeration remains an important microbiological task across clinical, pharmaceutical, food, and environmental settings, while AI-enabled image analysis offers tools to support counting and plate interpretation [1,8,9]. Manual reading is variable, labor-intensive, and difficult to audit at scale. Earlier automated approaches also struggled with overlap, atypical morphology, and background artifacts [19,26,27]. Deep learning allows models to learn variation in colony appearance rather than relying only on fixed thresholds [33,35,39]. Current evidence supports the feasibility of AI-assisted colony analysis, but performance remains closely dependent on culture format, organism morphology, imaging conditions, and validation design. Results obtained in one workflow should therefore not be assumed to transfer directly to another.

Published studies report the benefits of automated analysis in specific laboratory workflows, including systems integrated with TLA [36,39,54]. These findings should be interpreted according to the task and validation setting, rather than as evidence of a general improvement in CFU-counting accuracy. Studies of urine-culture triage and food-related image analysis illustrate the importance of aligning model outputs with domain-specific decision rules [4,39,43,49,76]. Task-specific combinations of complementary analytical functions, such as colony localization, instance separation, aggregate counting, and artifact handling, warrant further investigation. The workflows reviewed here support the further investigation of these combinations [36,65], while their added value must be established through comparison with strong single-model baselines, component ablation, and external validation. Figure 6 summarizes the authors’ conceptual proposals for matching methods to colony-analysis tasks.

The strength of the available evidence differs across tasks. The clinical studies discussed here provide evidence for plate-level triage and semi-quantitative interpretation that should not be transferred to direct CFU enumeration. Fungal image-classification studies likewise do not validate fungal colony counting. In pharmaceutical QC and cleanroom environmental monitoring, validation must address low-count specificity.

Future AI applications may extend beyond counting to other colony-analysis tasks. These include colony localization for automated picking, morphology-aware phenotyping, growth-pattern characterization, and preliminary identification from subtle texture and color cues [67,111,112]. Time-lapse imaging may further enable the analysis of growth dynamics such as growth rate, lag time, and spatio-temporal behavior. This could support earlier and more informative interpretation than endpoint reading alone [67,111,112]. Future studies should define the microbiological question first, specify the required model output, evaluate errors under relevant colony morphologies and culture formats, and report whether performance is preserved across independent plates, batches, organisms, imaging systems, or laboratories.

## Figures and Tables

**Table 2:** Public and semi-public datasets and benchmark resources for microbial colony-image analysis.

Dataset/Resource	Scale/Organisms	Annotation Type	Supported Tasks	Acquisition	Main Limitations	Availability; Notes	Ref.
AGAR; AGAR image dataset	18,000 high-resolution images; 336,442 annotated colonies; 5 microbial species	Colony-level bounding boxes and species labels; plate-level countability labels	Object detection, species classification, counting, benchmarking	Nikon D3500 camera, 4000 × 6000 pixels; IDS UI-5370CP camera, 2048 × 2048 pixels	Defined organism set and imaging conditions; transfer to other media or workflows requires validation	Publicly described resource	[60,61]
Makrai et al. annotated bacterial-colony dataset	369 images; 56,865 annotated colonies; 24 veterinary-relevant bacterial species	Colony-level bounding boxes and species labels	Object detection and colony localization	Smartphone imaging using LG Nexus 5X, iPhone 6, and HUAWEI P30 Lite.	Limited media and imaging diversity; not designed for all clinical, food, or pharmaceutical formats	Reusable dataset associated with the Scientific Data publication	[55]
Pawlowski et al. synthetic/style-transfer resource	Based on AGAR images and empty-plate backgrounds; 5 microbial species	Colony-level bounding boxes, class labels; and generated instance masks	Synthetic augmentation and detection/counting support	Synthetic images derived from AGAR; 512 × 512-pixel patches.	Synthetic data require validation on independent real images before deployment	Resource supports synthetic training-data generation rather than serving as a deployment benchmark	[56]
OpenFungi and related fungal image datasets	Macroscopic and microscopic fungal images; scale depends on dataset version; 5 fungal genera	Image-level labels for fungal recognition/classification	Fungal recognition and taxonomic classification	Macroscopic images: 4624 × 3472 pixels	Primarily supports classification, not validated colony enumeration or filamentous burden estimation	Useful as a morphology-recognition benchmark, not direct fungal-counting evidence	[62]

Note: equipment manufacturers: Nikon D3500 camera, Nikon Corporation (Tokyo, Japan); IDS UI-5370CP camera, IDS Imaging Development Systems GmbH (Obersulm, Germany); LG Nexus 5X, LG Electronics Inc. (Seoul, Republic of Korea); iPhone 6, Apple Inc. (Cupertino, CA, USA); HUAWEI P30 Lite, Huawei Technologies Co., Ltd. (Shenzhen, China).

**Table 3:** Non-comparative summary of representative AI studies relevant to colony analysis and enumeration.

Evidence Category	Study/System, Year	Dataset Size; Type	Primary Task; Output	Evaluated Output	Reported Metric(s)	Interpretation and Limitations	Ref.
A. Enumeration-oriented evidence and benchmark resources	Ferrari et al., 2017	28,500 blood-agar colony-segment images	Colony-segment cardinality classification; counting support	Colony-segment cardinality classification	Segment accuracy: CNN 92.1%; HCF 79.5%; watershed 69.0%. Per-colony error: 0.28; 0.80; 0.88, respectively.	Segment classification, not whole-plate agreement.	[33]
	Zhang et al., 2022	Five original images augmented to 960 samples; comparison evaluated on 10 images sampled from 60 new images	YOLO-based detection; counting support	Detection	Source-defined average accuracy (ACC): 97.4%; false-negative rate (FNR): 1.5%. See the table note for the ACC definition.	Detection metric, not ordinary classification accuracy or count agreement; limited-image evaluation.	[57]
	Cao et al., 2024	Plate-count agar and nutrient agar images	U^2^-Net + ResNet50 segmentation/counting pipeline	Counting (segmentation-based)	Overall recovery 97.82%. For positive reference counts, percentage recovery is 100 × (1—absolute count error/reference count).	For zero-colony crops, recovery is recall for the zero-colony class. Recovery alone does not show signed bias or per-plate limits of agreement.	[36]
	Shi et al., 2019	Food-sample pour plates; Luria–Bertani agar	Hyperspectral discrimination; counting support	Counting (multi-modal)	R^2^ = 0.9998 vs. manual count	R^2^ measures fit to manual counts; it does not exclude systematic bias or establish agreement or interchangeability.	[43]
	Wang et al., 2025; Colony-YOLO	310 images; 23,524 colonies	Microcolony object detection	Detection	mAP@0.5 96.1%	Detection mAP@0.5; not a measure of colony-counting accuracy.	[40]
	Makrai et al., 2023	369 images; 56,865 annotated colonies	Public bacterial-colony detection dataset	Detection (benchmark resource)	Dataset resource	Annotated benchmark resource	[55]
	Pawlowski et al., 2022	100 real images as base for synthetic generation	Synthetic data; augmentation	Detection and counting	Fully stylized training data, Faster R-CNN: mAP@[0.50:0.95] = 0.416; MAE = 4.49 colonies/plate image.	Real AGAR test images. MAE gives absolute count error, not bias or limits of agreement	[56]
	AGAR; Majchrowska et al., 2021	18,000 images; 336,442 annotated colonies	Public colony-image benchmark	Detection, classification (benchmark resource)	Dataset resource	Benchmark dataset; external generalization requires separate validation	[60,61]
B. Adjacent workflow-support evidence, not direct enumeration validation	APAS; Glasson et al., 2017	9224 urine samples	Automated urine-culture plate interpretation	Plate-level triage (semi-quantitative)	Blood agar: sensitivity 99.0%; specificity 84.5%.	Growth detection on blood agar in urine cultures; not exact colony counting.	[5]
	WASPLab; Faron et al., 2020	12,931 urine samples	Growth screening and semi-quantitation	Plate-level triage (semi-quantitative)	Sensitivity 99.8%; specificity 72.0%	Growth-screening performance under the study conditions; not sensitivity or specificity for exact colony counts.	[76]
	BacterioSight; Alouani et al., 2022	114,074 urine-culture images	Classification	Plate-level triage	Accuracy >97%; intra-institution AUC ≥0.98; cross-institution AUC ≥0.90	Plate classification and discrimination. AUC spans decision thresholds and does not measure count error or agreement.	[39]
	DeepColony; Signoroni et al., 2023	Digital microbiology culture-plate images	Hierarchical global plate interpretation	Plate-level triage	Workflow-level performance reported in source study	Hierarchical plate interpretation; no numerical counting-agreement result is summarized in this row.	[35]
	OpenFungi; Cighir et al., 2025	Macroscopic and microscopic fungal images	Fungal recognition; classification	Morphology classification	Accuracy: 99.79% (macroscopic images) and 97.82% (microscopic images)	Fungal image classification, not colony-number estimation. These accuracies do not validate fungal enumeration.	[62]

Note: A covers enumeration methods and datasets; B covers triage and morphology classification. Metrics retain source-specific definitions and evaluation units and cannot be ranked across rows. For Zhang et al. [57], average accuracy (ACC) is calculated as TP/(TP + FP + FN), with TN set to zero in that study. For Ferrari et al. [33], per-colony error is the square root of the sum of squared segment-count errors divided by the total reference colony count in the test set; it is not MAE. TP, true positives; FP, false positives; FN, false negatives; TN, true negatives; HCF, handcrafted features; ACC, average accuracy; FNR, false-negative rate; MAE, mean absolute error; mAP, mean average precision; IoU, intersection over union; AUC, area under the receiver operating characteristic curve.

**Table 4:** Sector-specific colony-analysis challenges, analytical approaches, and validation needs.

Sector	Example or Use	Challenge	Analytical Approach and Validation Need	References
Clinical microbiology	Urine culture; calibrated-loop inoculation	Irregular or swarming growth	Segmentation describes colony patterns; swarming may invalidate enumeration and requires a review or invalid-plate flag.	[72]
Clinical microbiology	Urine-culture plate reading	High colony count (>300 colonies per plate) or confluent growth	Evaluate plate-level triage separately from direct counting; do not infer exact counts for non-enumerable plates.	[33,35,39,76]
Clinical microbiology	Mixed growth and chromogenic media	Polymicrobial interpretation	Validate colony categories and presumptive identification for the medium and incubation conditions; these outputs do not establish exact CFU counts.	[35,77,78]
Food industry	Pour plate enumeration	Food debris	Hyperspectral discrimination separates colonies from matrix particles in the reported setting; transfer requires independent validation.	[43]
Food industry	Processing many culture plates	Throughput and auditability	Measure acquisition, analysis, correction, and reporting time; retain images and counting decisions for review.	[54,79]
Food industry	Standardized culture methods	Counting rules and compliance	Apply method-specific counting and sample-preparation rules; validate automated results against the reference procedure.	[1,80,81]
Food industry	Yeast and mold analysis in food cultures	Fungal enumeration	CNN-based analysis of macroscopic features supports fungal recognition and may inform future enumeration methods; direct evidence for validated fungal colony counting remains limited.	[62,82,83]
Pharmaceutical product monitoring	Microbial limits testing	Low counts and colony-like artifacts	Assess false positives and false negatives on low-count plates containing scratches, bubbles, or product residues.	[26,84]
Pharmaceutical product monitoring	Qualification of automated culture-reading methods	Equivalence to the reference procedure	Establish intended use, acceptance criteria, and product- and method-specific performance before routine implementation.	[85,86,87]
Pharmaceutical product monitoring	Image and result management	Traceability and data integrity	Retain images, review decisions, and result histories; qualify instrument and information-system interfaces.	[54,85,86,87]
Environmental monitoring	Cleanroom: contact and settle plates	Grade A no-growth requirement; detection at 0 versus ≥1 CFU	Evaluate false positives on negative plates and false negatives at one colony. APAS-specific reports provide preliminary workflow evidence.	[88,89,90]
Environmental monitoring	Water and air; membrane formats where applicable	Low-contrast filter backgrounds	Evaluate membrane-specific imaging and counting conventions; the cited monitoring literature is contextual rather than direct AI validation.	[9,16,91,92,93]

cGMP: current Good Manufacturing Practice.

**Table 5:** Minimum reporting and validation framework for AI-enabled colony analysis according to intended output.

Intended Output	Minimum Performance Reporting
Counting	MAE; MAPE where defined; signed bias by density stratum; CCC; Bland–Altman bias and limits of agreement
Object detection	mAP@0.5 and mAP@[0.5:0.95] separately; AP by object-size bin
Semantic or instance segmentation	Dice or IoU; for instances, AJI or Panoptic Quality
Plate triage	Sensitivity and specificity with 95% CIs; NPV at local prevalence; review-rate versus residual-risk curve
Calibration	Expected calibration error (ECE), with a reliability plot where decisions use confidence

Abbreviations: MAE, mean absolute error; MAPE, mean absolute percentage error; CCC, concordance correlation coefficient; AP, average precision; AJI, aggregated Jaccard index; NPV, negative predictive value; ECE, expected calibration error. MAPE is not defined when the reference count is zero.

## Data Availability

No new data were created or analyzed in this study. Data sharing is not applicable to this review. Figure 1b summarizes the Web of Science search described in Section 2.1. Figure 2b–e replots published numerical data from the studies identified in the caption. The culture-plate photographs in Figure 3, Figure 4, Figure 5 and Figure 6 were previously collected by the authors and are included solely for illustration.
